# Computational nanotechnology for functional coatings

**DOI:** 10.1186/1758-2946-3-S1-O24

**Published:** 2011-04-19

**Authors:** Welchy Leite Cavalcanti, Michael Hoffmann, Marc Amkreutz, Peter Schiffels

**Affiliations:** 1 Fraunhofer Institute for Manufacturing Technology and Advanced Materials in Bremen – IFAM, Bremen, D-28359, Germany

## 

The computer simulations are powerful tools to understand and determine properties and behaviour for a broad range of materials and diverse applications, going to different time and length scales. In the applied computational chemistry group at IFAM the computer simulations have been successfully applied to support the experiments and develop coatings with improved functionalities. The computational nanotechnology has been carried out to develop enhanced coatings with different functionalities, for instance, anti-acing function, anti-corrosion performance, antifouling, adhesion, and release properties.

In this context the molecular state and the interactions among the coating matrix, the particles and the surface play a fundamental rule. The computer simulations are an effective way to determine properties influenced by the atomistic/molecular state.

In this presentation we will provide an overview of successful cases where we have applied the computational nanotechnology to support the experiments and develop coatings with improved functionalities, as anti-corrosion, anti-icing and adhesion properties.
